# A case of corrosive esophagitis causing extensive cicatricial esophageal stenosis treated by esophageal bypass with supercharged pedicled jejunal pull-up

**DOI:** 10.1016/j.ijscr.2019.10.021

**Published:** 2019-10-17

**Authors:** Masaaki Saito, Hirokazu Kiyozaki, Tamotsu Obitsu, Erika Machida, Jun Takahashi, Iku Abe, Yuta Muto, Toshiki Rikiyama

**Affiliations:** Department of Surgery, Saitama Medical Center, Jichi Medical University, Saitama, Japan

**Keywords:** Alkali ingestion, Corrosive esophagitis, Pedicled jejunal bypass

## Abstract

**Introduction:**

Swallowing a corrosive substance causes delayed gastrointestinal stenosis due to scar formation. Here, we report on our use of esophageal bypass using a supercharged pedicled jejunal flap to treat cicatricial esophageal stenosis caused by corrosive esophagitis.

**Presentation of case:**

Nineteen years before presentation, a 57-year-old man had swallowed a chemical cleaning agent, which caused extensive corrosive cicatricial stenosis from the thoracic upper esophagus to the gastric fornix. An enterostomy had been created, and the patient had since been subsisting on enteral nutrition. However, he wanted to be able to eat through his mouth again and was referred to our department for treatment. With the exception of the cervical esophagus, circumferential cicatricial stenosis was present throughout the esophagus and gastric fornix, with severe adhesions to the surrounding tissue. It was decided not to perform esophagectomy but to perform esophageal bypass surgery using a supercharged pedicled jejunal flap.

**Discussion:**

Despite the extremely high risk of cancer in the stenotic esophagus due by corrosive esophagitis, indicating that esophagectomy should be performed if possible, we chose to perform bypass surgery because the severe adhesions posed a high risk of early injury to the surrounding organs.

**Conclusion:**

We suggest that esophageal bypass using pedicled jejunal pull-up “supercharging” by creating anastomoses between the jejunal and internal thoracic vessels is the optimal procedure for patients with extensive cicatricial esophageal stenosis caused by corrosive esophagitis.

## Highlights

• Swallowing corrosive substances leads to gastrointestinal stenosis due to scarification.
• Bypass surgery was performed as adhesions posed risk of injury to adjacent organs.
• Esophageal bypass with a “supercharged” pedicled jejunal flap was performed.
• The technique creates anastomoses between jejunal and internal thoracic vessels.
• It is an optimal technique for treatment of stenosis caused by corrosive esophagitis.

## 1 Introduction

Swallowing a corrosive substance (a strong acid or alkali) rapidly causes liquefactive necrosis down to the deep layers of the upper gastrointestinal tissue; thus, perforation may occur. Regardless of healing, delayed gastrointestinal stenosis due to scar formation is characteristic of this injury [1].

Gastrointestinal stenosis due to the swallowing of a corrosive substance can be treated using conservative therapies such as endoscopic balloon dilation if the extent of the stenosis is not too great as well as not too severe. However, if the stenosis is extensive, then the symptoms are unlikely to improve.

We treated a patient with extensive cicatricial esophageal stenosis caused by corrosive esophagitis after he swallowed an alkali. We performed esophageal bypass surgery using a pedicled jejunal flap that had been “supercharged” by anastomosis with the jejunal artery and vein and the right internal thoracic artery and vein to increase perfusion to the esophagojejunal anastomosis, with good results.

The work in this case has been reported in line with the SCARE criteria [2].

## 2 Presentation of case

A 57-year-old man had swallowed a strong alkaline cleaning agent in a suicide attempt 19 years previously. He was taken in an ambulance to a local hospital, where corrosive esophagitis and cicatricial esophageal stenosis were diagnosed. An enterostomy was created for long-term nutritional management. For the next 19 years, the patient had left the esophageal stenosis untreated and subsisted entirely on enteral nutrition. However, he wanted to be able to eat through his mouth again and was, therefore, referred to our department for treatment.

**Fig. 1:**
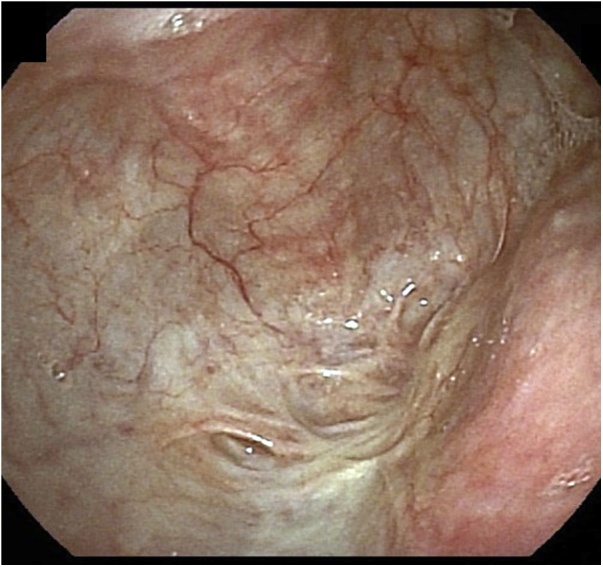
Upper gastrointestinal endoscopy showing the completely obstructed cervical esophagus.

**Fig. 2:**
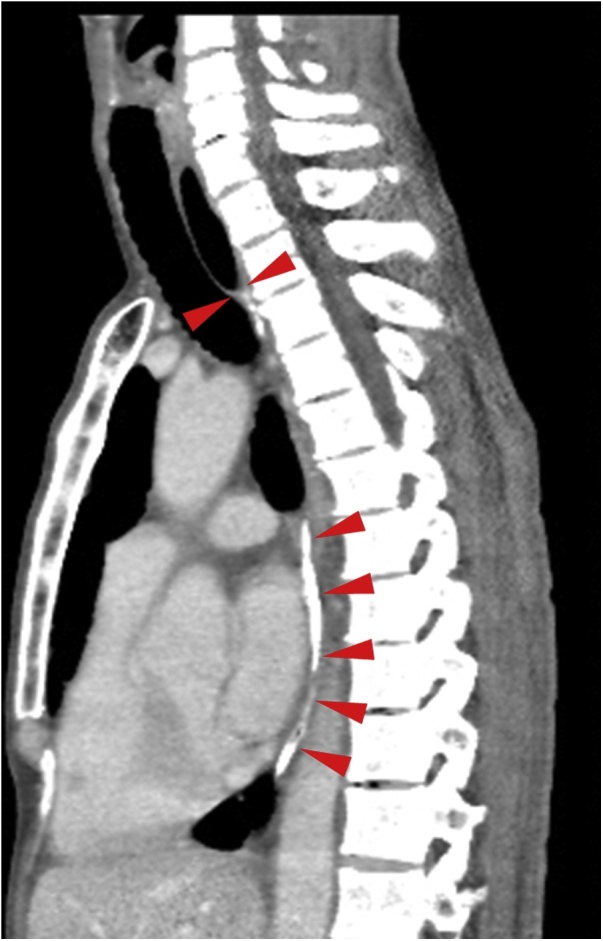
Thoracoabdominal CT (sagittal plane) showing a blind end formed by the cervical esophagus at the superior margin of the sternum and apparent scarring, stenosis, and calcification of the lumen of the thoracic esophagus (arrowhead: stenotic esophagus).

Upper gastrointestinal endoscopy showed that the cervical esophagus was scarred and completely obstructed, 25 cm from the incisors (Fig. 1). Contrast-enhanced computed tomography (CT) showed that the esophagus came to a blind end at the superior margin of the sternum, below which it was scarred and calcified (Fig. 2). There were no tumorous lesions in the esophagus.

**Fig. 3:**
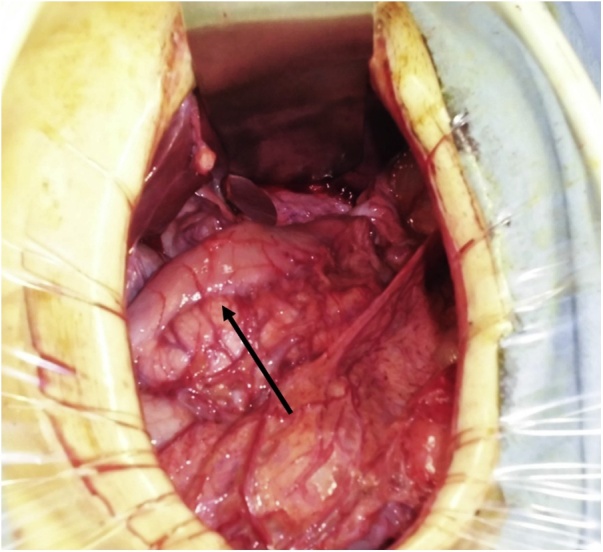
Cicatricial deformation and scarring of the stomach from the fornix to the gastric corpus (arrow: pyloric antrum).

**Fig. 4:**
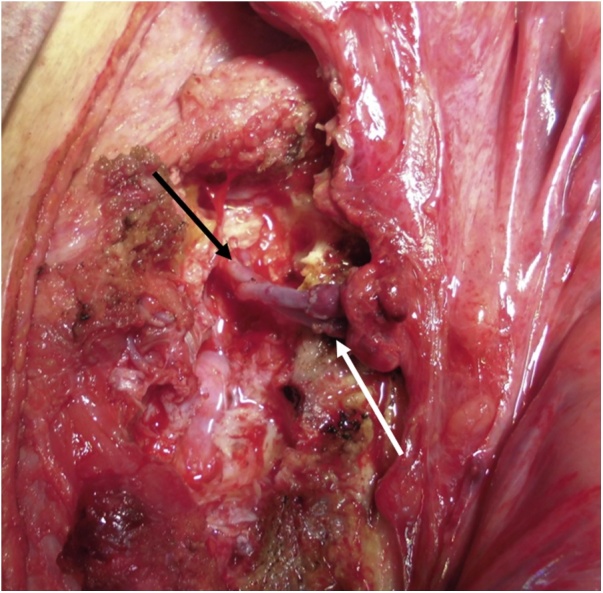
The pedicled jejunal flap is pulled up via the antethoracic route and an anastomosis with the cervical esophagus is created.

**Fig. 5:**
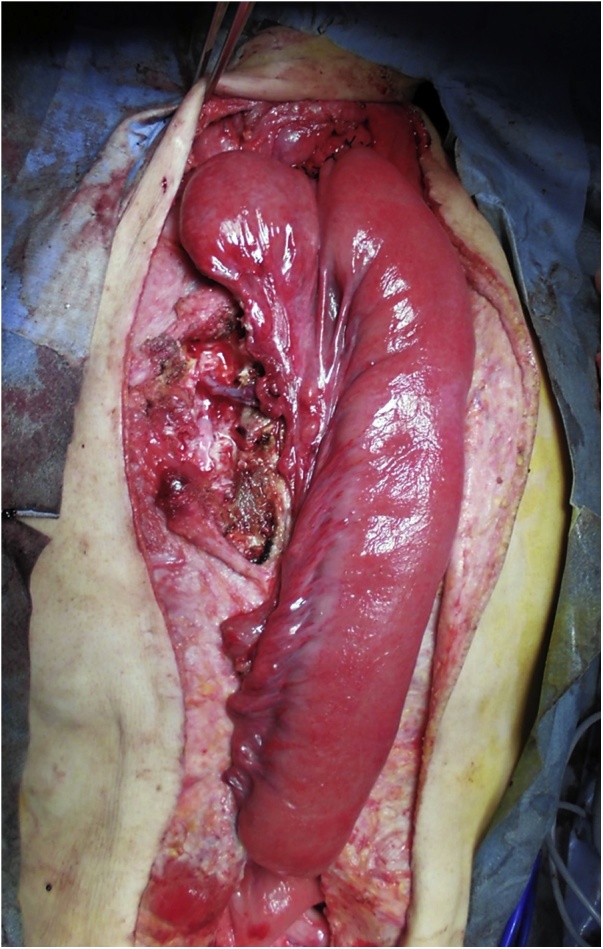
“Supercharging”: anastomoses are created between the internal thoracic artery and vein (black arrows) and the marginal artery and vein of the elevated jejunum (black arrows).

Laparotomy revealed cicatricial deformation and stenosis extending continuously from the abdominal esophagus and gastric fornix to the gastric corpus (Fig. 3). The thoracic esophagus in the mediastinum exhibited cicatricial stenosis, with severe adhesions to the surrounding organs; therefore, we decided to create a bypass to the cervical esophagus by jejunal pull-up, without performing esophageal and gastric resection. Observation via an incision in the neck above the level of the collar revealed that at the level of the superior margin of the clavicle, the esophageal tunica adventitia had turned white and the esophagus was completely occluded; the esophagus was resected at the oral side immediately above the site of obstruction. We decided to perform jejunal reconstruction, and the vessels supplying the elevated jejunum were identified. The jejunum was transected at the origin of the second and third jejunal arteries to create a pedicled jejunal flap, which was pulled up via the antethoracic route. The right second and third rib cartilages were resected, and the internal thoracic artery and vein were exposed for graft production. Anastomoses between the marginal artery and vein of the elevated jejunal flap and the internal thoracic artery and vein were created microsurgically using 10-0 nylon (the “supercharge” technique) (Fig. 4). The esophagojejunal anastomosis was created as an end-to-side anastomosis (Fig. 5).

Subsequently, the jejuno-jejunal anastomosis was created as an end-to-side anastomosis.

The surgery was completed by closing the neck incision; however, a portion of the skin was left open to provide a monitoring window for observing the blind end of the elevated jejunum. His postoperative course was smooth, and he was discharged on postoperative day 17. Three years postoperatively, outpatient monitoring has continued, but no stenosis of the anastomosis site or other symptoms have occurred.

## 3 Discussion

We herein reported our use of esophageal bypass with a supercharged pedicled jejunal flap to treat cicatricial esophageal stenosis caused by corrosive esophagitis in a 57-year-old man, with good postoperative results.

Corrosive esophagitis is usually due to esophageal injury caused by attempting suicide from swallowing a pharmacological substance, leading to esophageal ulceration or stenosis. The severity of the esophageal injury varies depending on the extent and severity of exposure to the pharmacological substance, as well as the type of substance involved (acid or alkali) [1]. The method of treatment also varies. Corrosive esophagitis starts with ulceration 1–3 weeks after exposure to the pharmacological agent and cicatricial stenosis from the granulation stage. Scarring gradually progresses until, in most cases, cicatricial stenosis is reportedly often complete more than 8 months later. The first-choice treatment for cicatricial stenosis is fluoroscopic or endoscopic dilation with a bougie, and it is considered safe to perform dilation 6–7 weeks after injury [3]. However, dilation may not be very effective in patients with extensive stenosis, and surgical treatments such as bypass surgery or esophagectomy are recommended in such cases [4]. Recent reports have also found that expandable metallic stents may be effective [5]. There are no clear criteria for the timing of surgical treatment, but many reports have stated that it should be delayed until around a year after the injury when cicatricial stenosis is complete [4]. This is because the extent of cicatricial stenosis cannot be identified any earlier, and there is a high risk of postoperative stenosis at the anastomosis site.

Inflammation that occurs in alkali-induced corrosive esophagitis extends into the deep layers of the esophagus and causes fibrosis, which often makes dissection difficult. Bypass surgery has the advantage of eliminating obstructions to the passage of food without the necessity of dissecting areas of severe fibrosis. However, if the esophagus is preserved, cancer may later develop in the residual esophageal tissue. Reportedly, it takes around 30 years for esophageal cancer to occur and the rate of carcinogenesis is 22 times that of healthy individuals [[6], [7], [8]]. The esophagus should, therefore, be removed, if possible.

Our patient had initially declined gastrointestinal reconstruction, only to decide 19 years later that he no longer wanted to subsist on enteral nutrition; hence, he requested surgery. Intraoperatively, cicatricial stenosis of the esophagus and stomach, and severe adhesions to the surrounding organs, were apparent. To avoid the risk of damaging the surrounding organs and reducing surgical invasiveness, it was decided to perform bypass surgery rather than esophagectomy. However, the risk of metachronous esophageal cancer developing in the area of cicatricial stenosis and inability to monitor the remaining stomach remained. The question of whether to prioritize the risks associated with esophagectomy or those associated with secondary cancer is a topic for future study.

The method of reconstruction and the organ used may involve the gastric tube, the ilio-colon, or the jejunum, either pulled up or as a free graft. In the present case, as preoperative colonoscopy to identify colon lesions could not be performed, we decided to perform reconstruction by jejunal pull-up. Although the use of the elevated jejunum as the reconstructive organ has the general advantages of simplicity and requiring few anastomoses, the difficulty of elevating becomes a problem. The second and third jejunal artery and vein are often transected to make its elevation easier, and the “supercharging” technique has frequently been used to secure perfusion to the far end of the elevated jejunum in this process, by creating anastomoses between the second and third jejunal vessels and the cervical or internal thoracic vessels [[9], [10], [11], [12]]. Hirabayashi et al. performed supercharged pedicled jejunal reconstruction in 12 cases and reported that anastomosis failure only occurred in the first two [13]. We also carried out “supercharging” in the present case by transecting the origins of the second and third jejunal artery and vein, enabling easy pull-up of the jejunum via the antethoracic route, and creating microsurgical anastomoses between the right internal thoracic artery and vein and the marginal artery and vein of the jejunum.

Despite the extremely high risk of cancer in the esophagus made stenotic by corrosive esophagitis, which means that esophagectomy should be performed if possible, we chose to perform bypass surgery as the severe adhesions posed a high risk of early injury to the surrounding organs. Considering the potential for secondary cancer, the preserved esophagus and stomach will require careful long-term follow-up using diagnostic imaging, such as CT and positron emission tomography-computed tomography (PET-CT).

## 4 Conclusion

We performed esophageal bypass using pedicled jejunal pull-up “supercharging” by creating anastomoses between the jejunal and internal thoracic vessels, with good postoperative results. We suggest that this is an optimal procedure for patients with extensive cicatricial esophageal stenosis caused by corrosive esophagitis.

## Declaration of Competing Interest

All authors declare that there is no conflict of interest.

## Funding

This research did not receive any specific grant from any funding agency in the public, commercial, or not-for-profit sectors.

## Ethical approval

The institutional ethics committee considers that ethical approval is not necessary for a case report.

## Consent

A written consent for the publication of this case report with accompanying images was obtained from the patient. Consent can be provided to the editors of this journal on request.

## Author contribution

Study conception and design: Saito, Obitsu.

Data acquisition and interpretation: Saito, Muto.

Surgery and patient follow-up: Saito, Kiyozaki, Obitsu.

Preparation of images: Machida, Takahashi, Abe.

Drafting the paper: Saito, Obitsu.

Critical revision: Kiyozaki, Rikiyama.

Final approval of submitting the manuscript: Saito, Machida, Abe, Muto, Kiyozaki, Rikiyama.

## Registration of research studies

researchregistry4857.

## Guarantor

Masaaki Saito, the corresponding author of this paper.

## Provenance and peer review

Not commissioned, externally peer-reviewed
